# Regional pattern and signatures of gut microbiota in rural residents with coronary heart disease: A metagenomic analysis

**DOI:** 10.3389/fcimb.2022.1007161

**Published:** 2022-11-28

**Authors:** Wenlong Li, Huijun Li, Shaolan Wang, Keyang Han, Yuan Liu, Zhen An, Hui Wu, Juan Li, Jie Song, Weidong Wu

**Affiliations:** ^1^ School of Public Health, Xinxiang Medical University, Xinxiang, Henan, China; ^2^ Institute of Infectious Disease Prevention and Control, Zhengzhou Center for Disease Control and Prevention, Zhengzhou, Henan, China

**Keywords:** microbial signatures, coronary heart disease, gut microbiota, rural residents, metagenomic analysis

## Abstract

Coronary heart disease (CHD) is tightly associated with gut microbiota, but microbiota heterogeneity limits the application of microbial biomarkers and personalized interventions demand regional-specific features. The purpose of this study was to comprehensively characterize the regional pattern of gut microbiota in rural residents with CHD and assess the predictive value and clinical correlations of local microbial signatures. We profiled the gut microbiota by shotgun metagenomic sequencing from 19 CHD and 19 healthy residents in rural Xinxiang, China, and tested the physiological parameters. The results indicated that microbial diversity, as well as KEGG orthology (KO) and carbohydrate-active enzymes (CAZymes) functions, deserved no significant disparities between CHD and healthy residents. The relative abundance of Bacteroidetes phylum was significantly lower and unclassified *Lachnospiraceae* genus, and *Eubacterium rectale* species were markedly higher in CHD residents compared with the healthy control. Co-occurrence network revealed a more diverse and scattered ecology in CHD residents. LEfSe identified 39 potential biomarkers and butanoate metabolism and glycosyltransferases families were the enhanced KO and CAZymes in CHD residents, respectively. Twenty key signatures were determined by the random forest algorithm and most of them belonged to the *Clostridium* cluster. These key signatures harbored a superior accuracy of 83.9% to distinguish CHD and healthy residents and, fasting serum insulin, diastolic blood pressure, and body mass index were the top three clinical parameters influencing the gut bacterial community. Furthermore, we also found that low-density lipoprotein and waist circumference had significantly positive correlations with the members of the *Clostridium* cluster. These findings expand our knowledge in the regional-specific pattern of gut microbiota for rural CHD residents and highlight the non-invasive diagnostic value and clinical correlations of microbial signatures.

## Introduction

Coronary heart disease (CHD), one of the major cardiovascular diseases, characterized by coronary artery stenosis and myocardial hypoxia, has become a worldwide public health challenge (Zhang et al., 2008; Zhou et al., 2019). Patients with CHD are generally represented by nontypical clinical symptoms, recurrent episodes of chest pain, or sudden heart attack, ultimately increasing potential health hazards and heavy social-economic burden. Despite substantial therapeutic interventions implemented to alleviate CHD risks, it remains the leading cause of morbidity and mortality globally (Bansilal et al., 2015).

Recently, substantial studies suggested that CHD patients existed gut dysbiosis, accompanied by structure, composition, or functional alterations of intestinal microbiota (Karlsson et al., 2012; Jie et al., 2017). Meanwhile, the metabolisms of gut microbiota such as short-chain fatty acids (SCFAs), secondary bile acids, and trimethylamine-N-oxide (TMAO), are also implicated in intestinal homeostasis and affect the atherosclerotic processes by mediating cholesterol metabolism, uric acid metabolism, oxidative stress, or inflammatory reactions (Trøseid et al., 2020). Gut microbiota had been regarded as a non-invasive diagnostic biomarker and conferred a potential target for preventing and treating CHD (He et al., 2018). However, as the key metabolic filter, gut microbiota always harbored a high dynamic ecology system and bore various environmental stress, thus causing disparities for the same disease phenotype. Moreover, microbiota heterogeneity largely limited applications of the healthy gut microbiome reference range and disease prediction models, thereby decreasing the accuracy of disease prediction (He et al., 2018). Geography variations were reported as the strongest factor affecting gut microbiota, beyond environmental elements and dietary patterns (He et al., 2018). Generally, geographical location, to some extent, constrained the entire variations of local environments, dietary habits, and lifestyles. Vangay et al. found that geographical migration was associated with immediate loss of gut microbiome diversity and function in which post-migration strains and functions displaced previous strains and functions (Vangay et al., 2018). Moreover, the gut microbiome was also in response to the geographical latitude, followed by a positive correlation between Firmicutes and a negative association for Bacteroidetes (Suzuki and Worobey, 2014). These evidence emphasis the influences of geography and demand personalized interventions for the human microbiome.

In China, with the development of industrialization and urbanization, the burden of CHD in rural areas is rising and has surpassed the urban districts (Ma et al., 2020). Ayeni et al. revealed that pristine fiber degraders and the low inter-individual variation were progressively lost with urbanization, thereby highlighting the disparities between rural and urban areas (Ayeni et al., 2018). Meanwhile, evidence had revealed that industrialization and urbanization decreased microbial exposure and contributed to the increased prevalence of non-communicable diseases (Selway et al., 2020), implying the unique features in rural areas. Therefore, we hypothesize that the regional pattern of gut microbiota deserves local-specific traits and harbors superior performance for disease prediction and precise prevention. Henan Province is located in central China and has a population of over 109 million in 2019, which is also the largest agricultural province, and nearly half of the population lives in rural areas. So, it is critical to decipher the regional specific traits and provide references for disease intervention by targeting microbiota. This study aimed to reveal the regional pattern of gut microbiota in rural residents with CHD, and assessed the value of local microbial signatures for CHD prediction.

## Material and methods

### Study participants

According to the urban-rural division codes of the national bureau of statistics of China, 19 rural residents with CHD alone and 19 healthy controls in Qiliying and Langgongmiao, Xinxiang county were recruited for this study. The inclusion criteria of CHD were as follows: (1) aged 45 to 79 years; (2) diagnosed with CHD by physicians in a secondary hospital; (3) written informed consent and willingness to participate in this study. Except for CHD, the subjects who had any other diseases (e.g., cerebrovascular diseases, metabolism diseases, gastrointestinal diseases, respiratory diseases, immunity diseases, cancers, or disease-related complications), who took antibiotic therapy or probiotics within three months, or who had cognitive impairment and cannot cooperate, were excluded. Moreover, the healthy controls had no history of chronic diseases, taking any medication, or severe lifestyle that might disrupt gut microbiota. Characteristics of all the participants including age, gender, health status, education level, marital status, occupations, monthly income, smoking, and drinking condition were collected by questionnaires. This study was approved by the Ethics Committee of Xinxiang Medical University for Human Studies (protocol number: XYLL-2016242) and all subjects gave written informed consent.

### Sample collection, physician examination and laboratory tests

Fresh fecal and blood serum samples were collected and extracted after fasting for at least eight hours and immediately frozen at -80°C. Height, weight, waist circumference (WC), hip circumference (HC), systolic blood pressure (SBP), diastolic blood pressure (DBP), and pulse rate (PR) were measured at least twice and the means were defined as final values, while red blood cells (RBC), white blood cells (WBC), Hemoglobin (Hb), platelet counts, alanine aminotransferase (ALT), aspartate aminotransferase (AST), Uric acid (UA), creatinine (Cre), fasting serum insulin (FSI), fasting blood glucose (FBG), glycosylated hemoglobin (HbA1c), total cholesterol (TC), triglyceride (TG), high-density lipoprotein (HDL), and low-density lipoprotein (LDL) were also tested. Body mass index (BMI) was calculated as weight in kilograms divided by height in meters squared.

### DNA extraction, quality control, library preparation and shotgun metagenomic sequencing

Bacterial DNA was extracted at Beijing Novogene Bioinformatics Technology Co., China, using the Cetyltrimethyl Ammonium Bromide/Sodium Dodecyl Sulfonate method. The purity and integrity of DNA were detected by agarose gel electrophoresis, and the concentration of DNA was quantified by Qubit fluorometry. Qualified DNA was fragmented by ultrasonic wave and prepared library using the NEBNext^®^ Ultra™ DNA kit. Then, Agilent 2100 Bioanalyzer was used to detect the fragments size and a quantitative polymerase chain reaction (qPCR) was performed to quantify the library concentration. Finally, eligible DNA libraries were sequenced on the Illumina HiSeq platform and raw data was obtained.

### Bioinformatics analysis

Raw fastq reads were quality-checked by Trim Galore and adapter, and low-quality reads and chimera sequences were removed. BMTagger (Rotmistrovsky and Agarwala, 2011) was used to remove the host genomic sequences against the human genome reference (hg38) and Fastqc qualified the clean data finally. Microbial diversity analysis was performed on QIIME platform (Caporaso et al., 2010) and the Kaiju program (Menzel et al., 2016) was used to assign taxonomic classification against NCBI RefSeq database. Genome assembled by the MEGAHIT tool (Li et al., 2015) and Prodial program (Hyatt et al., 2010) was used to predict open reading frame (ORF). For functional prediction, Diamond was utilized to align to KEGG orthology (KO) (Kanehisa et al., 2017) and carbohydrate-active enzymes (CAZymes) database (Lombard et al., 2014) and Salmon tool (Patro et al., 2017) was used to estimate the abundance of genes.

### Statistical analysis

For baseline characteristics of participants, categorical variables were represented by frequency and proportion, utilizing chi-square tests to compare their variations. Continuous variables were represented by the mean and standard deviation (SD) or median (M) and interquartile range (IQR) as appropriate. Wilcoxon rank-sum test was used to compare the differences of α-diversity between groups, whereas permutational multivariate analysis of variance (Adonis test) was applied for dimensionality reduction of two communities. Principal coordinate analysis (PCoA), based on Bray-Curtis distance metrics, was performed with the R vegan package to assess the β-diversity and functional alterations. Venn diagrams were used to demonstrate the differences in microbial composition and co-occurrence networks were established with Gephi software (Bastian et al., 2009) to decipher the ecologic pattern between groups. To reduce network complexity, those average relative abundances > 0.02% in each group could be selected to construct co-occurrence networks. Spearman’s correlation coefficient between two species was considered robust if the absolute r value > 0.8 with a corresponding ‘holm’ adjusted p-value <0.001. Linear discriminant analysis (LDA) effect size (LEfSe) (Segata et al., 2011) was performed to disclose the different microbiota and functions. Random forest models were utilized with the R Random Forest package (Breiman, 2001) to screen the signatures that distinguish CHD patients and healthy controls. Canonical correspondence analysis (CCA) was used to analyze the relationships between the bacterial community and altered physiological indicators. All analyses were completed using the R software, and a p-value < 0.05 in a two-tailed test was considered statistically significant.

## Results

### Characteristics of participants

Baseline characteristics of 38 subjects were shown in **Table 1**. Compared to the control group, the level of LDL, WC, HC, SBP, DBP, and BMI were significantly higher in CHD group, while the distribution of FSI, ALT, AST, and TG between groups were markedly different (p-value < 0.05).

**Table 1:** Baseline characteristics of the control and CHD groups.

Variables	Control (n = 19)	CHD (n = 19)
Age (years)	57.74 ± 9.48	62.63 ± 7.08
Gender (male/female)	9/10	8/11
Education (≤primary school/≥ middle school)	6/13	12/7
Marital status (married/divorce or widow)	16/3	18/1
Occupation (farmer/worker/other)	8/5/6	4/4/11
Monthly income (<500/500~1000/≥1000)	8/5/6	3/10/6
Smoking (yes/no)	5/14	6/13
Drinking (yes/no)	3/16	5/14
WC (cm)	78.52 ± 5.95	92.91 ± 7.60^***^
HC (cm)	94.62 ± 4.63	100.55 ± 4.69^***^
BMI (kg/m^2^)	22.91 ± 2.13	27.19 ± 3.03^***^
SBP (mmHg)	115.26 ± 9.20	132.28 ± 12.59^***^
DBP (mmHg)	73.11 ± 6.19	80.04 ± 8.37^**^
PR (bpm)	72.16 ± 7.94	74.58 ± 10.93
WBC, 10^9^/L	5.29 ± 1.40	5.44 ± 0.85
RBC, 10^12^/L	4.69 ± 0.45	4.72 ± 0.40
Platelet, 10^9^/L	248.00 ± 69.82	217.21 ± 49.25
Hb (g/L)	136.53 ± 14.76	143.58 ± 12.65
UA (μmol/L)	273.95 ± 63.23	314.69 ± 98.89
TC (mmol/L)	4.76 ± 0.64	5.25 ± 1.19
LDL (mmol/L)	2.64 ± 0.51	3.09 ± 0.71^*^
HDL (mmol/L)	1.35 ± 0.26	1.28 ± 0.25
FBG (mmol/L)	5.27 ± 0.45	5.57 ± 0.71
HbA1c (%)	5.28 ± 0.48	5.54 ± 0.54
Cre (μmol/L), M (IQR)	64 (12.5)	60 (13)
FSI (mmol/L), M (IQR)	4.4 (1.7)	7.3 (4.95) ^**^
ALT (U/L), M (IQR)	18 (6.5)	23.46 (9) ^*^
AST (U/L), M (IQR)	21 (3)	24 (7.84) ^*^
TG (mmol/L), M (IQR)	0.94 (0.345)	1.38 (1.25) ^*^

Values are presented as mean ± SD or median and interquartile range (IQR). All p-values are from the Welch’s t test or the Wilcoxon rank sum test, except for gender, education, marital status, occupation, monthly income, smoking, and drinking history (χ^2^ tests or Fisher’s exact test). ^*^p-value < 0.05; ^**^p-value < 0.01; ^***^p-value < 0.001 vs. Control. WC, waist circumference; HC, hip circumference;BMI, body mass index; SBP,systolic blood pressure, DBP, diastolic blood pressure; PR, pulse rate; RBC, red blood cells; WBC, white blood cells; Hb, Hemoglobin; UA, Uric acid; TC,total cholesterol;LDL, low-density lipoprotein; HDL, high-density lipoprotein; FBG, fasting blood glucose; HbA1c, glycosylated hemoglobin; Cre, creatinine; FSI, fasting serum insulin; ALT, alanine aminotransferase; AST, aspartate aminotransferase; TG, triglyceride.

### Diversity and taxonomic profiles of the gut microbiota

**Figure 1:**
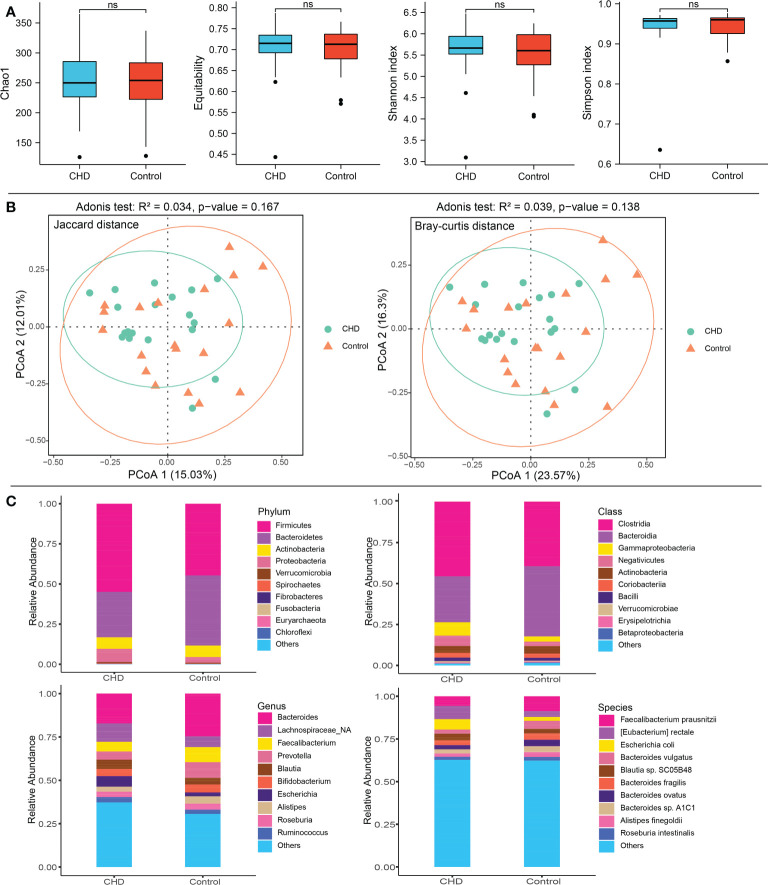
Diversity and taxonomic composition of gut microbiota in CHD and control groups. **(A)** α-diversity including Chao1, equitability, shannon and simpson index. **(B)** β-diversity and PCoA based on Jaccard and Bray-Curtis distance metrics. **(C)** Taxonomic composition of gut microbiota at phylum, class, genus, and species levels between CHD and control groups. ns, no significant.

After rarefying to a specific sequencing depth, α- and β-diversity were calculated. α-diversity encompassing the chao1, equitability, shannon, and simpson indices, was displayed in **Figure 1A** and demonstrated no significant difference between groups (p-value > 0.05). For β-diversity, estimated by Jaccard and Bray-Curtis distance, PCoA showed that the gut microbiota in CHD group relatively clustered together, whereas those in the control group were relatively scattered (**Figure 1B**). The contribution of PCoA1 and PCoA2 was 15.03% and 12.01% for Jaccard metrics, while Bray-Curtis metrics demonstrated by 23.57% and 16.3%, respectively. Adonis test indicated that gut microbiota displayed no significant differences between groups (Jaccard: R^2^ = 0.034, p-value = 0.167; Bray-Curtis: R^2^ = 0.039, p-value = 0.138).

Aligning to the non-redundant protein database (NCBI RefSeq), 21 bacterial phyla, 42 classes, 304 genera, and 655 species were identified. The top 10 phyla, classes, genera and species were shown in **Figure 1C**. Firmicutes (CHD vs. Control: 54.91% vs. 44.72%), Bacteroidetes (CHD vs. Control: 28.35% vs. 43.61%), Actinobacteria (CHD vs. Control: 7.13% vs. 7.21%), and Proteobacteria (CHD vs. Control: 8.27% vs. 3.55%) were the top four dominant taxa at the phylum level. The top three bacterial classes were Clostridia (CHD vs. Control: 45.62% vs. 39.45%), Bacteroidia (CHD vs. Control: 28.07% vs. 42.86%), and Gammaproteobacteria (CHD vs. Control: 8.13% vs. 3.11%). The top three bacterial genera were Bacteroides (CHD vs. Control: 17.22% vs. 24.68%), unclassified Lachnospiraceae (CHD vs. Control: 10.56% vs. 6.15%) and Faecalibacterium (CHD vs. Control: 5.63% vs. 8.70%), while the top three species were *Faecalibacterium prausnitzii* (CHD vs. Control: 5.63% vs. 8.70%, p-value = 0.052), *Eubacterium rectale* (CHD vs. Control: 7.77% vs. 3.44%), and *Escherichia coli* (CHD vs. Control: 6.08% vs. 2.22%, p-value = 0.298). The mean relative abundance of Bacteroidetes phylum (p-value = 0.048) was lower in CHD group, whereas unclassified Lachnospiraceae genus (p-value = 0.017), and *E. rectale* species (p-value = 0.006) displayed higher relative abundances.

### Functional profiles of the gut microbiota

**Figure 2:**
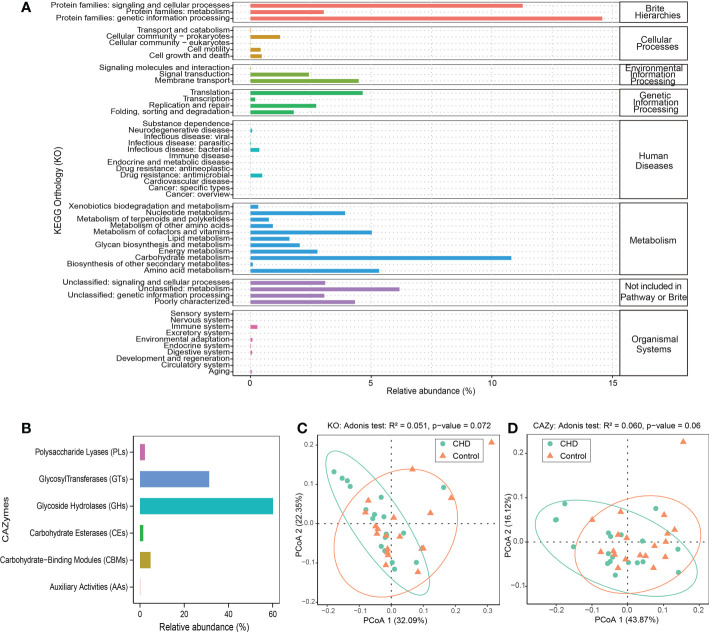
Function profiles of gut microbiota. **(A)** KEGG orthology (KO) relative abundance of gut microbiota. **(B)** carbohydrate-active enzymes (CAZymes) relative abundance of gut microbiota. **(C, D)** Dimensionality reduction PCoA and Adonis test of KO and CAZymes based on Bray-Curtis distance metrics between CHD and control groups.

KO and CAZymes database were aligned to explore the functional characteristics of gut microbiota. Genetic information processing, cellular community (prokaryotes), membrane transport, translation, drug resistance (antimicrobial), carbohydrate metabolism, unclassified metabolism, and immune system were the most abundant functions at level 2 of each KO hierarchy (**Figure 2A**), respectively. Glycoside hydrolases (GHs) and glycosyl transferases (GTs) were the enriched CAZymes and the relative abundance was more than 90% (**Figure 2B**). The dimensionality reduction PCoA, both KO and CAZymes, showed that the gut microbiota in the control group clustered together, whereas those in the CHD group were relatively scattered. The contribution of PCoA1 and PCoA2 was 32.09% and 22.35% for KO, while demonstrated by 43.87% and 16.12% for CAZymes, respectively. Adonis test showed that KO and CAZymes of gut microbiota between CHD and control groups were no significant differences (Adonis test p-value >0.05, **Figures 2C, D**).

### Species variations and co-occurrence network analysis of gut microbiota

**Figure 3:**
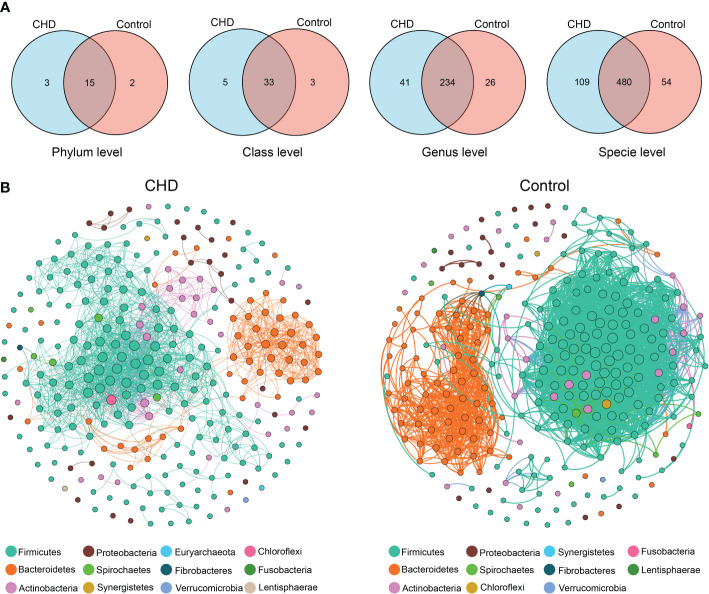
Species variations and co-occurrence networks between CHD and control groups. **(A)** Venn diagrams of species variations at phylum, class, genus, and species level. **(B)** Co-occurrent networks in CHD and control groups. Links denote significant (‘holm’ adjust p-value < 0.001 and Spearman’s absolute r > 0.8) correlations with ‘holm’ adjust and weight by each correlation. The nodes in network are colored according to phylum and weight by the node degree of each species.

To identify the species variations and co-occurrence pattern of gut microbiota, Venn diagrams (**Figure 3A**) were illustrated and co-occurrence networks (**Figure 3B**) were established. At the phylum level, 15 phyla were shared in both two groups, with 3 and 2 independent phyla in CHD and control, respectively. At the class level, there were 33 common genera, with 5 and 3 independent genera in CHD and control, respectively. At the genus level, there were 234 common genera, with 41 and 26 independent genera in CHD and control, respectively. At the species level, there were 480 common species, with 109 and 54 independent species in CHD and control, respectively. The co-occurrent network in CHD group consisted of 299 nodes and 1635 edges, while the network in control group harbored 299 nodes and 3727 edges. The control group had a more complex connected network: the average path length, clustering coefficient, and modularity index were followed by 4.201, 0.71, and 0.355, while in CHD group demonstrated 2.819, 0.659, and 0.546, respectively.

### Gut microbiota Alterations and identification of microbial signatures

**Figure 4:**
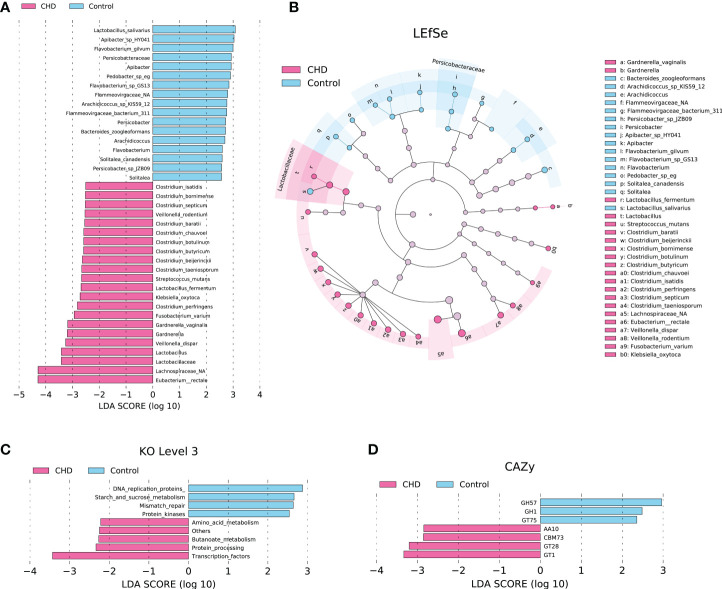
Differential taxa, KOs, and CAZymes between CHD and control groups identified by linear discriminant analysis (LDA) effect size (LEfSe) with default parameters (p-value < 0.05 and LDA scores > 2.0). **(A)** Differential taxa identified by LDA score. **(B)** Cladogram of differential taxa between CHD and control groups. **(C)** Differential KOs at level 3 identified by LEfSe. **(D)** Differential CAZymes identified by LEfSe.

To probe the alterations of gut microbiota between CHD and control groups, LEfSe with default parameters (p-value < 0.05 and LDA scores > 2.0) identified 39 taxa as potential biomarkers (**Figures 4A, B**) and 9 KOs (**Figure 4C**), as well as 7 CAZymes (**Figure 4D**), were detected as differential functions. These differential taxa are mainly enriched in Lactobacillaceae and Persicobacteraceae families, and the *Clostridium* cluster. DNA replication proteins, starch and sucrose metabolism, mismatch repair, and protein kinases were enhanced functions in control group, while amino acid metabolism, butanoate metabolism, protein processing, and transcription factors were increased in CHD group. For CAZymes functions, glycoside hydrolases (GH) 57, GH1, and glycosyltransferases (GT) 75 families such as β-glucosidase, α-amylase, and self-glucosylating β-glucosyltransferase were enhanced in control group, while auxiliary activities (AA) 10, carbohydrate-binding modules (CBM) 73, GT28, and GT1 families such as copper-dependent lytic polysaccharide monooxygenases, chitin-associated modules of residues, 1,2-diacylglycerol 3-β-galactosyltransferase, and glucuronosyltransferase were elevated in CHD group.

**Figure 5:**
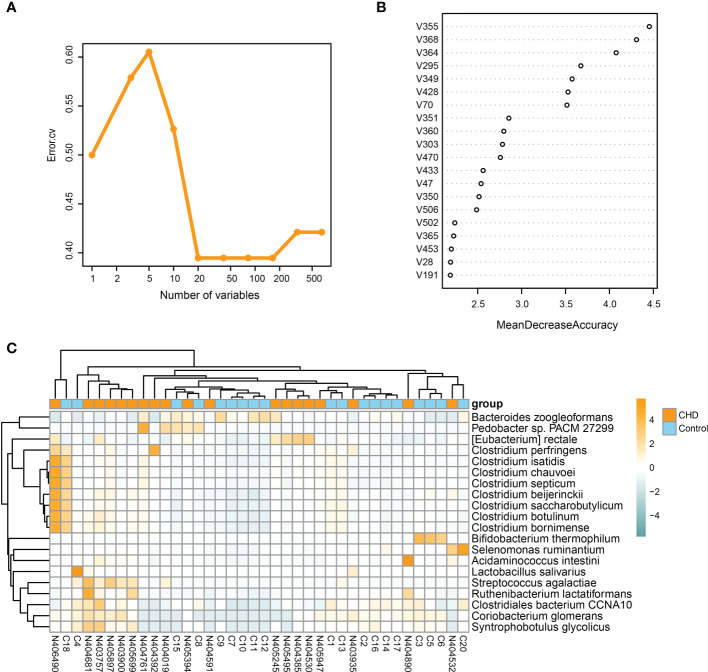
Identification of key signatures between CHD and control groups. **(A)** 10-fold cross-validation by random forest method identifies twenty key signatures. **(B)** Mean decrease accuracy of species in the random forest model. **(C)** Relative abundance of twenty key signatures illustrated by heatmap.

To explore the key species distinguishing the CHD and control groups, a random forest algorithm was further performed to determine the key microbial signatures. By applying 10-fold cross-validation on the random forest model, twenty microbial species were selected based on the lowest mean error rate (**Figure 5A**). Thus, we selected the top twenty candidates based on the mean decrease accuracy as the key signatures (**Figure 5B**). The relative abundance of these key signatures was displayed by the heatmap in **Figure 5C**. These key signatures demonstrated higher relative abundance in the CHD group, except for *Lactobacillus salivarius, Bacteroides zoogleoformans, Clostridiales bacterium CCNA10, Selenomonas ruminantium,* and *Bifidobacterium thermophilum*.

### Microbial prediction value and clinical associations

**Figure 6:**
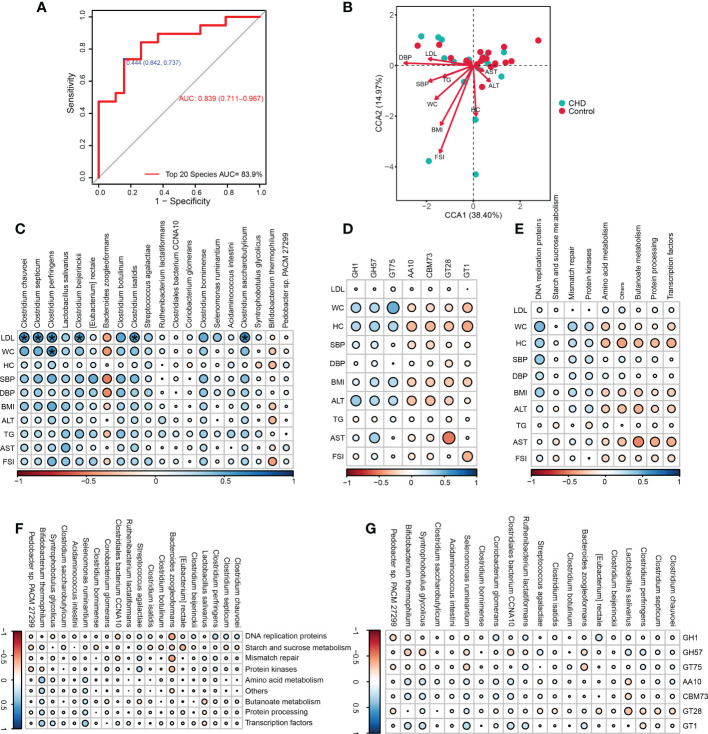
Microbial prediction value and clinical associations of key signatures. **(A)** The receiver operating characteristic (ROC) curve of the key signatures for CHD prediction. **(B)** Canonical correspondence analysis (CCA) between the bacterial community and altered physiological indicators. **(C-E)** Correlations among altered physiological parameters, key signatures, differential KOs and CAZymes. **(F, G)** Correlations among key signatures, differential KOs and CAZymes. ^*^ ‘holm’ adjust p-value < 0.05. WC, waist circumference; HC, hip circumference;BMI, body mass index; SBP,systolic blood pressure, DBP, diastolic blood pressure; LDL, low-density lipoprotein; FSI, fasting serum insulin; ALT, alanine aminotransferase; AST, aspartate aminotransferase; TG, triglyceride; AUC, the area under the ROC curve.

To determine the predictive capability of key signatures, we performed the receiver operating characteristic (ROC) curve and calculated the area under the ROC curve (AUC), as illustrated in **Figure 6A**. The sensitivity was 73.7%, and the specificity was 84.2%, with an AUC of 83.9% (95% confidence interval: 0.711-0.967). CCA showed that, with the gradient of clinical indicators, CCA1 and CCA2 explained 38.40% and 14.97% variations of gut microbiota between groups, respectively (**Figure 6B**). FSI, DBP, and BMI were the top three clinical parameters influencing the gut bacterial community, thereby implicated in the microbial alterations between groups.

Furthermore, the associations among key signatures, altered clinical parameters, differential KOs, and CAZymes functions were assessed. LDL was positively associated with *Clostridium* clusters including *Clostridium chauvoei, Clostridium septicum, Clostridium perfringens, Clostridium beijerinckii, Clostridium isatidis,* and *Clostridium saccharobutylicum,* while WC was also positively correlated with *C.perfringens* (**Figure 6C**). These altered clinical parameters had no significant correlations with those changed KOs and CAZymes (**Figures 6D, E**). Accordingly, key signatures also had no significant correlations with altered KOs and CAZymes (**Figures 6F, G**).

## Discussion

Gut microbiota is a complex, evolution-molded, and high dynamic ecological system, which plays important physiological roles in the human intestine and greatly extends the tolerance and regulation of the intestine to external stimulation and exposure (Santoro et al., 2018). Although the relationships between gut microbiota and CHD have been identified by substantial studies, alterations of microbial communities remain discord. These microbiota heterogeneities are largely attributed to geography variations, and local traits of gut microbiota could specifically affect the progress of CHD, thus modeling the regional microbial pattern (Fontana et al., 2019). Herein, we utilized the metagenomic sequencing method to comprehensively characterize the regional pattern of gut microbiota and evaluate the predictive value for CHD diagnosis.

The diversity of gut microbiota reflected the species richness in certain ecology and their disparities or space distance in different ecological niches. Numerous studies reported that gut dysbiosis was associated with alterations of microbial diversity, even though these results demonstrated inconsistency. Cui et al. found that α-diversity in CHD patients was significantly higher than that of the healthy controls (Cui et al., 2017), but the results of Zhu et al. and Toya et al. demonstrated the richness of gut microbiota was markedly decreased in CHD group (Zhu et al., 2018; Toya et al., 2020). However, Liu et al. assessed gut microbiota changes among CHD subgroups and indicated that the severity of CHD was associated with microbial diversity, including α- and β-diversity (Liu et al., 2019). For details, the evidence showed that no significant differences were found between healthy subjects and stable CHD, whereas the unstable angina group exhibited higher microbial diversity than the healthy control group. In our present study, we found that no disparities existed in microbial diversity between CHD and control groups. Although PCoA displayed rather closer distances within CHD samples, the similarity between CHD and control groups remained higher, reflecting the insignificant diversity of gut microbiota as well. Furthermore, Venn diagrams captured more microbial species in CHD group from the phylum to species level and the co-occurrence network in CHD demonstrated a higher modularity index, which implied that gut microbiota structure altered and tended to diverse and scattered modularity.

In accordance with previous studies (Cui et al., 2017; Toya et al., 2020), at the microbial taxonomic level, we found Firmicutes, Bacteroidetes, Actinobacteria, and Proteobacteria were the dominant taxa. The ratio of Firmicutes/Bacteroidetes (F/B) was elevated in CHD subjects, followed by 1.9 in CHD and 0.9 in healthy controls. The abundance of Bacteroidetes phylum was significantly decreased, whereas unclassified Lachnospiraceae genus and *E. rectale* species, belonging to the Firmicutes phylum, were markedly increased. The altered abundance of Firmicutes and Bacteroidetes implied the imbalance of competition stability and resulting potential function changes. Some researchers proposed that the change in F/B ratio could represent the dysbiosis of gut microbiota, but due to the methodological differences, poor characterization of the study population, and insufficient consideration of lifestyle-associated factors, it remained difficult to determine the F/B ratio as the marker for the dysbiosis of gut microbiota (Magne et al., 2020). The Lachnospiraceae family belonged to the Clostridial cluster XIVa of the phylum Firmicutes, and some members displayed strong hydrolyzing activities and short-chain fatty acids (SCFAs)-producing abilities (Vacca et al., 2020). Reversely, the increase of Lachnospiraceae was also associated with metabolic diseases such as chronic kidney diseases and diabetes, representing the potential of inflammation promotion (Qin et al., 2012; Vaziri et al., 2013). *E. rectale* was the butyrate-producing species and represented anti-inflammation properties by mediating the immunity of regulatory T cells (Furusawa et al., 2013; Fu et al., 2019). Previous studies found that *E. rectale* was depletion in CHD and heart failure patients (Kamo et al., 2017; Zhu et al., 2018), but we observed an increased relative abundance in CHD subjects. We inferred that the subjects in our study were tended to stable CHD patients and the depletion of *E. rectale* could be the final result of atherosclerosis or the individuals with severe CHD. Besides, the elevated relative abundance of *E. rectale* could also be the regional microbial feature in rural Xinxiang county.

Dispersal, local diversification, environmental selection, ecological drift, and co-evolution were regarded as the fundamental processes of community formation by ecological theory (Costello et al., 2012). The human gut microbiome could rapidly adapt to external environmental stress and intestinal microenvironment alterations through recombination *via* horizontal gene transfer (Smillie et al., 2011). Previous studies indicated that transport of simple sugars (phosphotransferase systems), amino acids, propanoate metabolism, lipopolysaccharide biosynthesis proteins, and tryptophan metabolism were enhanced in CHD patients, while virulence factors were also increased and the potential for synthesis of butyrate was decreased (Jie et al., 2017; Zhu et al., 2018). Our study found that the KO hierarchy of environmental information processing, human diseases, metabolism, and the organismal system was enriched in membrane transport, antimicrobial drug resistance, carbohydrate metabolism, and immune system at level 2 of KO. At level 3 of KO, we found the differential functions were mainly enhanced in amino acid and butanoate metabolism in CHD group, whereas starch and sucrose metabolism and genetic information processing were elevated in healthy controls. We considered that the enhanced butanoate metabolism in CHD subjects could be the response to gut dysbiosis and was demanded its anti-inflammation properties. In addition, gut microbiota encoded substantial CAZymes and played an essential role in the degradation of dietary glycans, thus providing biological energy for the human body (El Kaoutari et al., 2013). Our results demonstrated that GHs and GTs were the dominant CAZymes in rural Xinxiang and, GHs and GTs were enriched in healthy controls and CHD subjects, respectively. GHs cleaved glycosidic bonds by the insertion of a water molecule (hydrolysis) and the function of GTs was catalyzing glycosylation reactions by mediating glycosidic bond formations between sugar moieties and important biomolecules (Lombard et al., 2010; Koropatkin et al., 2012). These disparities reflected that the capability of degrading dietary polysaccharides was decreased and biosynthesis of large or small molecules was enhanced in CHD patients, probably affected by the different dietary patterns. Although PCoA and Adonis test displayed no significant differences for KO and CAZymes functions in both CHD and control groups, alterations could not be neglected.

LEfSe identified 39 differential taxa as potential biomarkers and most of them were enriched in species level between CHD patients and healthy controls, of which *E. rectale* and *L.salivarius* were the most significant species in CHD and control groups, respectively. We further identified twenty key signatures between groups with 10-fold cross-validation, based on the mean decrease accuracy of the random forest algorithm. We found that twelve of the twenty key signatures belonged to the Clostridia class, including *E. rectale, C. perfringens, C. isatidis, C. chauvoei, C. septicum, C. beijerinckii, C. saccharobutylicum, Clostridium botulinum, Clostridium bornimense, Ruthenibacterium lactatiformans, C. bacterium CCNA10,* and *Syntrophobotulus glycolicus*. Except for *C. bacterium CCNA10,* all of these Clostridia cluster species deserved higher relative abundances in CHD patients. Among them, *E. rectale, C. saccharobutylicum,* and *C. bornimense* were reported as butyrate-producing bacteria with beneficial effects (Tomazetto et al., 2016; Fu et al., 2019), whereas *C. perfringens, C. chauvoei, C. septicum, C. botulinum* were regarded as pathogenic bacteria (Ohtani and Shimizu, 2016; Rychener et al., 2017; Aldape et al., 2018; Pernu et al., 2020). Furthermore, *C. beijerinckii,* and *R. lactatiformans* plausibly had the potential to produce butyrate and lactate (Shkoporov et al., 2016; Seo et al., 2017), while the evidence about *C. isatidis, C.bacterium CCNA10,* and *S. glycolicus* remained less known. These findings were partly different from previous studies (Kamo et al., 2017; Kummen et al., 2018; Zhu et al., 2018), in which we showed that butyrate-producing bacteria displayed higher relative abundances than healthy population instead of representing lower richness or depletion. We considered that it could be explained by the followings: firstly, as we mentioned before, the severity of CHD in different pathological conditions would represent by differently gut ecology and the depletion of butyrate-producing bacteria was a gradual process; secondly, these identified key signatures in our study also consisted of pathogenic and beneficial Clostridium and the competition of them could result in rescue effects to the atherosclerotic phenotype; thirdly, the increased relative abundance of butyrate-producing bacteria could associate with the regulation of immunity in the intestine, in which gut dysbiosis reversely promoted the increase of butyrate-producing bacteria and mediated release of anti-inflammatory cytokines. Therefore, we still believed that, in the gut of CHD residents, these microbiota alterations tended to be negative effects rather than beneficial value.

Coronary angiography was the gold standard for CHD diagnosis, generally accompanied by invasive physical pain and heavy psychological burden, urgently demanding new non-invasive methods to conquer these disadvantages and microbial signatures were emerging. In this study, we also evaluated the diagnosis value of key signatures and the relationships between gut microbiota and clinical indicators. Our results demonstrated that these key signatures harbored superior discrimination and predictive value with a high AUC of 83.9%, exceeding the accuracy of 67.71% in the study of Zhu et al. with the subjects recruited in a Shanghai hospital and approaching the results of Jie et al. in a metagenome-wide association study (Jie et al., 2017; Zhu et al., 2018). With the constraint of altered clinical indices, most of the clinical indices departed from the healthy samples and possessed positive correlations with CHD samples, indicating gut microbiota in different groups was altered by these physiological parameters. Nevertheless, we also assessed the associations among altered clinical indices, key signatures, differential KO, and CAZymes. With the ‘holm’ adjust method, we only found a strong positive correlation among Clostridia cluster species, LDL, and WC, validating the essential role of the Clostridia cluster and emphasizing its alteration in the regional microbial pattern. Although no disparities were observed among key signatures, KOs, and CAZymes in our study, Liu et al. found that fecal and serum lipopolysaccharide had negative correlations with metabolic pathways (Liu et al., 2020), indicating that the level of lipopolysaccharide could be a sensitive indicator for functional changes, and new functional-associated biomarkers still need excavation and discovery. The mechanisms of host-microbiota interaction and the methods for CHD prevention by targeting gut microbiota remain to be explored.

However, there also exist some limitations. Firstly, the diagnosis of CHD was dependent on the medical history and the severity of CHD, as well as local dietary pattern, could not be evaluated, plausibly leading to information bias. Secondly, although we utilized metagenomic sequencing to explore the features of gut microbiota, the sample size remained small. Finally, the CHD predictive model was not assessed in an external cohort to further verify its general applicability. Therefore, future studies should further identify the application value of the regional microbiota predictive model and explore internal mechanisms and clinical translation of gut microbiota.

## Conclusion

In this study, we comprehensively characterized the regional pattern of gut microbiota in the rural population of Xinxiang county, China, and assessed the prediction value, as well as clinical associations. This evidence expands our knowledge in the regional-specific pattern of gut microbiota for rural CHD residents and highlights the potentiality of Clostridium cluster in non-invasive diagnosis and microbiota-targeting intervention.

## Data availability statement

The data presented in the study are deposited in the GSA database of CNCB-NGDC, accession number PRJCA012852, CRA008953.

## Ethics statement

The studies involving human participants were reviewed and approved by the Ethics Committee of Xinxiang Medical University for Human Studies (protocol number: XYLL-2016242). The patients/participants provided their written informed consent to participate in this study.

## Author contributions

WL: Investigation, Methodology, Data curation, Validation, Formal analysis, Visualization, Writing – original draft. HL: Investigation, Methodology, Data curation, Analysis, Validation. SW: Investigation, Data curation, Analysis, Validation. KH: Investigation, Data curation, Analysis, Validation. YL: Investigation, Data curation, Analysis, Validation. ZA: Investigation, Methodology, Data curation, Analysis, Validation. HW: Investigation, Methodology, Data curation, Analysis, Validation. JL: Investigation, Methodology, Data curation, Analysis, Validation. JS: Investigation, Methodology, Data curation, Analysis, Validation. WW: Conceptualization, Funding acquisition, Supervision, Writing - review & editing, Project administration, Resources, Validation. The authors read and approved the final manuscript.

## Funding

This work was supported by the National Natural Science Foundation of China (2016YFC0900803; 2017YFD0400301; 81961128031) and Innovation Research Program for Postgraduates of the Xinxiang Medical University (YJSCX201927Z).

## Conflict of interest

The author declares that the research was conducted in the absence of any commercial or financial relationships that could be construed as a potential conflict of interest.

## Publisher’s note

All claims expressed in this article are solely those of the authors and do not necessarily represent those of their affiliated organizations, or those of the publisher, the editors and the reviewers. Any product that may be evaluated in this article, or claim that may be made by its manufacturer, is not guaranteed or endorsed by the publisher.
